# Supplementary material for: Potato (Solanum tuberosum) as a New Host for Pentastiridius leporinus (Hemiptera: Cixiidae) and Candidatus Arsenophonus Phytopathogenicus
Source: Insects. 2023 Mar 13;14(3):281. doi: 10.3390/insects14030281 (PMC10058626; doi:10.3390/insects14030281)
Supplement: Supplementary file 1 [file insects-14-00281-s001.zip › insects-2248745-supplementary.pdf]

Table S1. Morphometric measurements and developmental stages of field-collected *Pentastiridius leporinus* nymphs on potato plants in September 2022.

	Length [µm]		Head width [µm]		
**Stage**	**Range**	**Mean ± SD**	**Range**	**Mean ± SD**	**n**
Third instar	1709–2502	2164±247	457–591	546±51	9
Fourth instar	1923–3895	2904±386	601–818	682±47	57
Fifth instar	3195–4926	3867±444	847–1080	944±69	34

Most were fourth-instar nymphs, as reported in previous studies [6]. To confirm the ability of *P. leporinus* to feed, oviposit and hatch on potato, adults were maintained on potato plants for oviposition and freshly hatched nymphs were measured. The five individual first-stage nymphs, measured shortly after hatching, had a head width of 156–264 µm, the mean width was 209.8 µm, and the length ranged from 491 to 921 µm (mean = 658 µm).

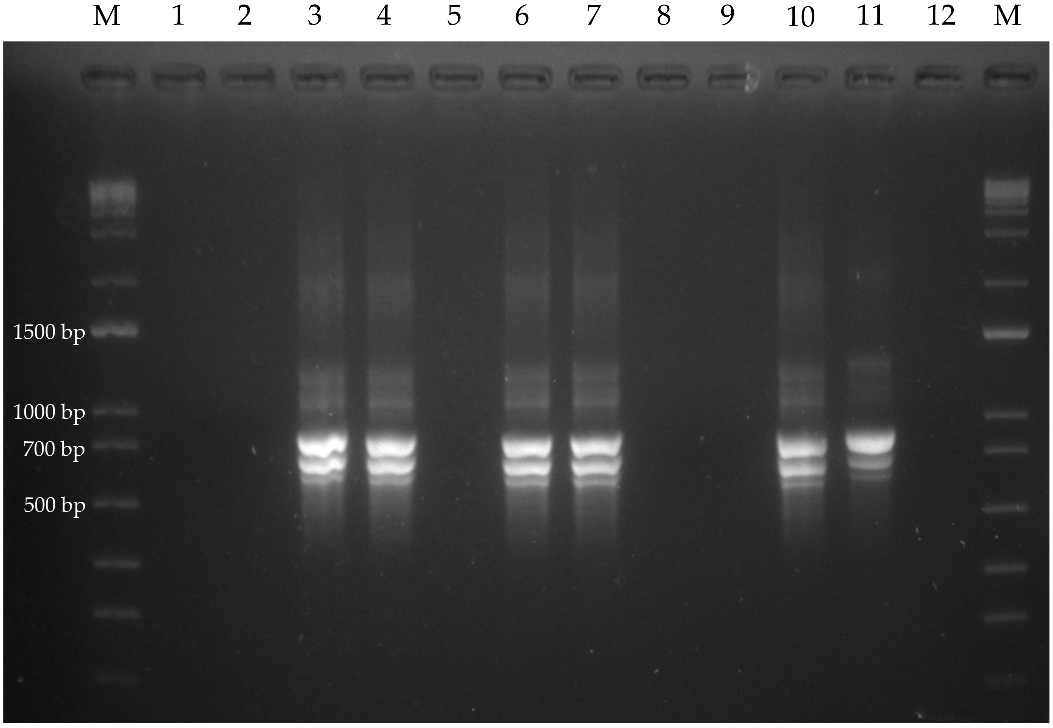

Figure S1. Gel electrophoresis of the nested PCR products obtained with primer Pairs Fra5/L1r and Alb1/Oliv1. 1 & 2: Negative tested (qRT-PCR) *Pentastiridius leporinus* from field, 3 & 4: Positive tested (qRT-PCR) *P. leporinus* from field, 5: Negative tested (qRT-PCR) sugar beet from greenhouse, 6 & 7 Positive tested (qRT-PCR) sugar beet from field, 8 & 9: Negative tested (qRT-PCR) potato from field, 10 & 11: Positive tested (qRT-PCR) potato from field, 12: Non-template-control, M: GenRuler 1 kb Plus DNA-Ladder (Thermo Scientific).
